# Exploring disaster preparedness in an obstetric unit in a district hospital in the Western Cape Province

**DOI:** 10.1186/s12913-024-11104-x

**Published:** 2024-05-21

**Authors:** Carla Horn, Nokwanda Edith Bam, Molekodi Jacob Matsipane

**Affiliations:** 1 NuMIQ Focus Area, Faculty of Health Sciences, North-West University, Potchefstroom Campus, Potchefstroom, South Africa; 2 NuMIQ Focus Area, Faculty of Health Sciences, North-West University, Mahikeng Campus, Mahikeng, South Africa

**Keywords:** Disaster, Emergency, Maternal, Midwives, Neonatal, Nurses, Obstetric, Pandemic, Preparedness, Public, Response

## Abstract

**Background:**

Research on disaster preparedness in public hospitals is limited, and specialised units such as obstetric departments need to be even more prepared when rendering health care to vulnerable populations. Disasters can be natural, such as floods due to human interventions, sinkholes due to mining, or pandemic occurrences, such as the recent COVID-19 pandemic. Research on disaster preparedness is limited, and even more so in specialised units such as obstetrics and evacuating a ward of maternal and neonatal patients present unique challenges. Being prepared for any disaster is the only assurance of effective patient healthcare during a disaster. This study explored and described nurses’ knowledge and attitudes regarding preparedness for a disaster in an obstetric unit in a public institution. The study aimed to make recommendations to improve disaster preparedness in an obstetric ward based on the nurses’ knowledge and attitudes.

**Methods:**

This study utilised an exploratory, descriptive qualitative design within a contextual approach. The data were acquired through individual interviews that were done using a semi-structured interview schedule. An observational walkabout was performed with the unit manager to validate interviewee responses. The study employed purposive sampling with a sample size of 17 nurses (*N* = 32, *n* = 17) and a response rate of 53%. The interviews were transcribed verbatim, and later, the data underwent analysis using theme analysis and a co-coder.

**Results:**

The results indicate that the participants demonstrate an awareness of disaster terminology but need more assertiveness in executing the institutional disaster policy. The results illustrate that more frequent training, disaster rehearsals, and simulations should be implemented to improve disaster readiness. Strategies are recommended to enhance preparedness for a disaster in the obstetric unit.

**Conclusion:**

The study findings recommend more education and training opportunities that should be regularly instilled as a practice within the obstetric ward. More disaster drills and simulation exercises should be performed to ensure confidence in disaster preparedness. Obstetric staff of all levels should be involved with policymaking and disaster plan development.

**Supplementary Information:**

The online version contains supplementary material available at 10.1186/s12913-024-11104-x.

## Background

A disaster refers to an event that disrupts the typical circumstances of living beings and results in a degree of suffering that surpasses the afflicted community’s ability to adapt to the immediate circumstances [1]. Ortiz-Barrios et al. [2] explained that disasters cause disruptions in healthcare units as the facilities get flooded with a surge of patients who may need immediate attention. Farah et al. [3] assert that disasters are escalating globally, with the African continent and Sub-Saharan Africa contributing to most disasters due to human-made events and natural forces. Conflicts, unsafe food and water, chemical and radiation incidents, building collapses, transportation incidents, a lack of water and power, air pollution, antimicrobial resistance, and the effects of climate change are also classified as disasters because they cause a sudden influx of deaths [4, 5].

In South Africa, disasters like strikes, power outages, water shortages, violent outbreaks, and disease outbreaks are likely to happen. A disaster is defined as a serious disruption of the functioning of a community or a society at any scale due to hazardous events interacting with conditions of exposure, vulnerability and capacity, leading to one or more of the following: human, material, economic and environmental losses and impacts [6]. The effect of the disaster can be immediate and localised but is often widespread and could last for a long period of time [6]. The effect may test or exceed the capacity of a community or society to cope using its own resources, and therefore may require assistance from external sources, which could include neighbouring jurisdictions or those at the national or international levels [6] The definition of a disaster contrasts with that of an emergency “an actual or imminent event or threatening condition that requires urgent action” [7]. All these happenings may harm the obstetric population, which can cause a surge in obstetric patient healthcare. A disruption or insufficiency in the functional healthcare system of the obstetric population might contribute to higher mortality and morbidity rates. According to Sahoo et al. [8], humanitarian crises, such as pandemics and disasters, result in an unparalleled disruption of healthcare services. In developing countries, such as South Africa, the occurrence of the maternal death rate 100 000 live births was 105.9 in 2019 and 88 in 2020 [8]. This mortality rate can be expected to be much higher in the occurrence of a disaster, and even more so in countries which are not prepared for disaster.

Disasters are unpredictable and may strike a population at any moment, so hospitals should always be prepared to manage a disaster. Disaster preparedness does not start with the response when disaster strikes but with thorough preparation beforehand. Ayenew et al. [4, 5] refer to the four stages of disaster planning: preparation, mitigation, response, and recovery. The study of Samsuddin et al. [9] indicates that disaster preparedness is a multi-faceted approach taken by the hospital’s stakeholders in terms of planning, organizing, knowledge training, equipping, exercising, evaluating, and taking corrective actions to prepare, reduce the effects of disaster, and ensure effective coordination during disaster response. Effective disaster management includes the proper planning and preparation of healthcare professionals. Nurses are the primary carers in a hospital setting before, during, and after a disaster. This includes utilizing skills, proper training, education, and knowledge to minimize harm and hazards to patients in their care [10].

The World Health Organization (WHO) classifies pregnant women as an important subgroup of the population that is most vulnerable in the event of a pandemic or a disaster [11]. Additionally, pregnant women have a 1.5-fold greater likelihood of requiring admission to a critical care unit in comparison to their non-pregnant counterparts [11]. Pregnant women are at high risk for health complications resulting from disasters such as low birth weight neonates, preterm births, miscarriages, maternal and neonatal infections, pre-eclamptic emergencies, and fetal distress, leading to increased rates of emergency caesarean Sect [12]. Therefore, an unexpected disaster may lead to an increased intake of hospitalized obstetric patients and may exceed the surge capacities of obstetric wards.

International research and scientific knowledge on disaster preparedness in obstetric units is limited, and only some reflect the conditions of governmental obstetric units within South Africa. This is concerning, considering that 87% of South Africa’s live births occur in government institutions and hospitals [13]. It is also known that 83% of the entire South African population receives public health care [14]. These statistics indicate that not only will 83% of the South African population be rushed to government hospitals during a disaster, but that 87% of obstetric cases will flood governmental hospitals in need of health care.

The context of the disaster within South Africa and the study setting may refer to exceeding surge capacity. Ayenew et al. [4, 5] refer to exceeding surge capacity as when the quantity of patients presenting within a given time exceeds the emergency center’s or healthcare center’s capacity to offer care without assistance. This can be expected during a surge of obstetric patients in public facilities, as 87% of the South African population depends on public healthcare. Considering the context above, disaster preparedness is vital in obstetric wards as vulnerable populations—both maternal and neonatal—have an increased need for disaster preparedness, and they will flood hospitals during a disaster.

The World Health Organization’s sustainable goal of reducing maternal mortality [15] cannot be achieved if obstetric health care is not optimized in South Africa, which entails effective disaster preparedness. The global statistics indicate that 99% of maternal deaths occur in low-and-middle-income countries (such as South Africa), where the majority (76%) occur in Sub-Saharan Africa [15]. The primary factor contributing to the elevated death rate is predominantly attributed to disparities in the availability and accessibility of fundamental healthcare services. The South African Maternal, Perinatal and Neonatal Health Policy (MPNH) stipulates that emergency preparedness, including epidemic, pandemic, and humanitarian situations, should be implemented for safe and effective obstetric health care [16]. The policy states that this can be achieved by strengthening the maternal, perinatal, and neonatal services’ capacity for emergency preparedness at all levels, including human resources, medical supplies, equipment, risk communication, and emergency management. The DoH emphasizes that pregnancy and birth are unpredictable, as are disasters [16], and thus urges facilities to be prepared to manage unexpected emergencies and to refer to a higher level of expertise when needed. South Africa’s National Department of Health [16] says that obstetric services are necessary for this high-risk group of people during disasters, epidemics, and pandemics. This highlights the need for disaster preparedness in obstetric wards in government hospitals.

This research study explores and assesses disaster preparedness in the obstetric wards of government hospitals in South Africa. This includes the exploration of nurses’ knowledge and attitudes toward disaster preparedness, general disaster management plans, policies, and documentation, competencies such as evacuation drills, and overall recommendations that can be made to improve disaster preparedness. Disaster and evacuation training is also to be considered. This research paper aims to contribute to the limited body of scientific knowledge regarding disaster preparedness within South Africa by making recommendations and, more specifically, focusing on obstetrics, which is even more limited. The study strives to make recommendations to improve disaster preparedness within obstetric units of South African hospitals to ensure more preparedness nationwide.

## Methods

The researcher embarked on a contextual, exploratory, descriptive qualitative (EDQ) research design within a qualitative research approach to explore nurses’ preparedness for disaster in an obstetric unit. This researcher utilized semi-structured individual interviews as a means of data collection. The researcher also used observational notes during the interviews and a walkabout tour through the obstetric unit. This enabled the researcher to validate the findings of what the participants said during the interviews.

The study focused on hazardous events, including emergencies, disasters, and high-impact events which can significantly disrupt hospital functioning and does not focus on medical emergencies relating to obstetrics.

### Study context

This study was conducted within an obstetric ward of a medium-sized, district-level government hospital within the Western Cape. The obstetric division includes neonatology, antepartum, intrapartum, postpartum and a Kangaroo-Mother-Care unit (KMC). The neonatal division has a capacity of eight neonatal beds, six closed incubators for in-stay neonatal patients and two open incubators, which are used for emergencies. The obstetric unit comprises a postnatal division and an antenatal division. The antenatal division includes seven antenatal beds and has two separate delivery rooms. The postnatal division contains eight maternal beds with 12 cribs for the neonates. The KMC division can admit 12 mothers with their infants. The total capacity of the obstetric unit can accommodate 61 patients in total. In case of overflow can another eight mothers and their infants be accommodated on sleeper couches. Thus, making the ultimate surge capacity of the unit 76 patients.

Sharma and Rani [17] explain that the recommended nurse-to-patient ratio is 1:4 within a specialized ward. Considering the maximum surge capacity of nurses and the nurse-to-patient ratio (1:10), there is a great need for this obstetric ward to be ready for the unforeseen disaster that may strike. During a state of disaster, it would thus be expected for one nurse to evacuate and manage ten patients if no staff shortages exist. The unit’s nursing staff comprises three to four professional nurses operating the antenatal and postnatal divisions. Only three professional registered nurses are on duty at night. Two enrolled nurses and one auxiliary nurse operate the nursery. One enrolled nurse operates the KMC division. This accounts for 30 to 32 nurses when all four shifts are included.

### Study population: participants

The study population referred to all the midwives and nurses who worked in an obstetric unit in the Western Cape during data collection. The target population of this study included and referred to the midwives and other category nurses working within the obstetric unit of a selected district government hospital in the Western Cape. The nurses included all categories: professional nurses, enrolled nurses, and nursing assistants.

The obstetric unit’s nursing staff comprises three to four professional nurses in antenatal and postnatal divisions. At night, only three professional nurses were on duty. Two enrolled nurses and one auxiliary nurse covered the nursery, and one enrolled nurse worked in the KMC division. The nurses included the night and day shift staff teams; the target population consisted of 32 people. In total 17 participants were interviewed.

### Sampling and sample size

The researcher employed a non-probable judgement sampling technique to elicit information-rich participant data. The target sample size of this study was 32 nurses (all categories) who worked at the select obstetric unit that met the inclusion and exclusion criteria. The researcher used data saturation as a guide to complete sampling (sampling of participants ended after data saturation was achieved). Data saturation is a concept elucidated by Saunders et al. [18], wherein they posit that the researcher reaches a point where no further data are being discovered, hence impeding the development of additional qualities. The researcher continued to interview participants until saturation was met. After the 17th participant was interviewed, no new information emerged. According to the co-coder, data saturation was reached after the 12th interview. Moreover, the researcher noted repetition and recurrence of sentences during the last few interviews and identical responses from participants. The sample size for this study thus consisted of 17 participants who participated in the open-ended, semi-structured interviews.

### Selection criteria and recruitment of participants

The selection criteria for this study were rigorously established to ensure that the participants would have the ability to respond effectively to the research questions. Furthermore, it was anticipated that the selection criteria for the sample population would be exact, as they influenced the data collected and the research study’s conclusions.

#### Inclusion criteria

The following were the inclusion criteria for participants to be included in the study.

- Permanently employed nurses: All levels of midwives and nurses that were registered with the South African Nursing Council (SANC). These include professional nurses, enrolled nurses, and enrolled nursing assistants. All levels of nurses may not have the same approach to disaster management, and therefore, all categories were included as they could have contributed meaningfully to the study.
- Nurses with experience of at least one year and more: The nurses included in this study had to be trained and orientated with a reasonable number of years of experience working in their respective wards.
- Nurses working in an obstetric unit and Neonatal Intensive Care Units (NICU): The study site was the obstetric unit, which included the antenatal, postnatal, and labour rooms. The NICU was also included as part of the obstetric unit as it is susceptible to fires due to flammable equipment.
- Kangaroo Mother Care area (KMC area) also had to be included as these are essential areas for evacuation during disaster since these locations have highly flammable gasses, and it is challenging to mobilize vulnerable populations, women postpartum that may be in pain and sickly neonates. KMC’s walls and building consist of cardboard building material and are also more susceptible to fire.

#### The exclusion criteria were

- Any participant who was on treatment for a mental illness, as participation may elevate fear if they felt unprepared to deal with a disaster.

### Recruitment

The specific hospital of the study was selected as a district hospital of a large surface area, receiving referrals of obstetric cases of more than ten facilities. The obstetric unit is expected to be prepared for a wide range of obstetric emergencies, complications and a large patient turnover. All of which these conditions are expected to worsen under the circumstance of a disaster. The researcher completed her community service at this hospital and, therefore, recruited an independent mediator to explain the study to the possible participants. The mediator assisted with recruiting participants, obtaining informed consent and other administrative tasks. Participants were recruited from all the divisions of the obstetric unit. The mediator informed the participants verbally about the research topic and the study. Once particpants met the inclusion and exclusion criteria, an information leaflet containing all the information about the study was handed to the potential participants to read. They were allowed to ask questions if any clarification was required. The participants were given 24 h to consider participating in the research project and were allowed to discuss participation with their families. Upon the acquisition of the duly signed informed consent papers, the participant was apprised of the forthcoming communication from the researcher, whereby the purpose would be to coordinate the scheduling of the interview at a mutually convenient time and day.

### Data collection process and collection tool

Data was collected via semi-structured interviews and observation. The researcher performed an observational walkabout with the unit manager to validate the responses and findings of the interviewed participants. The researcher requested the Nursing Manager to allocate her a vacant office within the obstetric unit to conduct the interviews. Field notes were taken during the interviews to capture body language, facial expressions, and any other data necessary that would not be available on the audio recordings. To validate the information gained from the participants, the researcher used observational data collection and field notes in the natural setting of the obstetric unit. During the walkabout, the researcher meticulously made field notes of everything she considered to be related to disaster preparedness.

The researcher used a validated tool to measure disaster preparedness during the walkabout in the obstetric unit with the unit manager. The framework to measure disaster preparedness was previously successfully used and described by Stander [19]. The researcher adapted the semi-structured interview questionnaire tool used and described by Moabi [20]. Questions 1 to 21 of the interview tool were previously published by Moabi [20]. Permission was granted by the author to allow the researcher to use the questions [1–4, 8–24] in the study. Questions 22 to 31 were drafted by the researcher with specific reference to obstetrics and neonatology. Some of the questions include the inquiry of past disaster experiences in the obstetric unit and response efforts. The complete interview guide can be found as “additional file 1” to this study. The researcher’s field notes recorded gestures of non-verbal communication of the participants during the interviews. The participants’ facial expressions, tone of voice, body language, reactions and emotions were recorded. The mediator recruited participants from all four shifts of nursing staff, two-night shift and two-day shift teams.

### Data analysis

Thematic analysis was the data analysis method utilized for this research study. Thematic analysis, according to Caulfield [21], involves analysing data, where the researcher examines the data to identify common themes, topics, ideas, opinions, knowledge responses, and patterns of meaning that come up continually. Braun and Clarke [22] refer to thematic analysis as a method of identifying, analysing, and reporting patterns (themes) within data. The analysis of the collected data and information was based on Braun and Clarke’s [22] six-step framework for analysing qualitative data as seen below:

The researcher followed a six-step process: familiarization with the data, initial coding, searching, and generating themes, reviewing themes, defining, and naming themes, and writing up the findings. The researcher immersed herself in the data set and followed the repeated reading of the data [22] with the transcriptions and typing of the interviews as step one (familiarization with the data). Generation of the initial codes of the data was done by the researcher by working systematically through the data set and paying special attention to repeated patterns and repeated responses of the participants. A professional co-coder assisted the researcher throughout the data analysis process. The researcher and co-coder performed data analysis separately and identified themes and subthemes. This was done to ensure control of analysis and accuracy, correlation of similarities, and clarification of differences within the results. The researcher and co-coder both worked together to define and name themes (phase five) [22].

## Results

Saturation of data was reached after seventeen participants were interviewed. The interviews were transcribed, and a co-coder was consulted to assist with the thematic analysis. Of the seventeen participants, nine were professional nurses, three enrolled nurses, and five were nursing assistants. Three of these nurses had one to five years of nursing experience, one nurse had five to ten years of experience, and five nurses had experience of ten to twenty years, eight of the nurses had more than twenty years of nursing experience. In regards to experience at the selected hospital, seven of the nurses had one to five years of experience, two nurses had five to ten years at the hospital, five of the nurses had ten to twenty years at the hospital, and three of the nurses had more than twenty years of experience at the selected hospital. Some of the participants verbalised experience of an actual live disaster occurrence during years of experience at the selected hospital such as the roof collapsing and a fire breakout. All seventeen interviews were coded and were included in the analysis and results. The thematic analysis yielded five themes with respective sub-themes.

### Theme 1: nurses knowledge regarding disaster preparedness

The nurses working in obstetrics display an elementary knowledge of disaster preparedness. The sub-themes encompass the comprehension of nurses regarding disaster management and their awareness of their tasks and obligations in the context of a disaster, as elaborated upon in the subsequent discussion.

#### Sub-theme 1.1: nurses’ understanding of disaster management

Participants demonstrate a clear understanding of what a disaster is. They verbalize that a disaster is something unexpected that happens suddenly and causes specific emotions, situations, and losses, such as a fire, flood, or gas explosion. The following participant quotes can strengthen this:

P2: *“Disaster, it’s a, happening that causes a lot of sadness, unhappiness, people get hurt, can get hurt through that. And it causes a lot of havoc*.”

P8: “*Something that is not like within the norm as your normal routine and something happen where it’s like threatening for you. Like a flood? Fire maybe.*”

Participants agreed that a disaster plan is a plan that must be put into action to manage the unknown, as evident in the following quotes:

P7: “*It’s a plan that you make to save your patients and your staff’s lives when a disaster should happen.*”

P8: “*Disaster plan is something that you put in place to do or when anything does happen that you know what to do.*”

Although few participants demonstrated awareness (participants 2, 3, 7) of the disaster plan’s location and content, most participants revealed feelings of uncertainty (participants 1, 5, 8, 9, 10, 11, 12, 14) regarding the disaster plan. The following can support this:

P5: *“I think we do have. But it’s not visible for us.”*

P8: *“Our disaster plan is supposed to be in the office.”*

P10: *“I don’t know because it’s not known to me. Since the plan is not known to me, I don’t know my position or when something like that happened.”*

#### Sub-theme 1.2: nurses’ understanding of their roles and responsibilities during a disaster

Participants [1, 3, 8, 10, 11] within the obstetric ward demonstrated an awareness of disaster drills and acknowledged their roles and responsibilities during a disaster drill. This is echoed in the following statements:

*P7: “That is when you practice what to do in case of a disaster, to make sure that your staff knows, and your patients know.”*

*P8: “Where you do preparation to prepare the staff. What they should do if they are fully equipped, if they are fully prepared for a disaster and if they have the knowledge and to know what to do, where to go to what.”*

Some participants [1, 2, 4, 10, 11] were aware and informed of their roles and responsibilities during a disaster drill, while others lacked assertiveness (participants 5, 10, 15). This is evidenced in the following quotes:

P8: “We have different functions because we have a different sections.”

P5: “Some of us don’t even know what is our roles.”

Disaster preparedness requires nurses to be prepared and ready for a disaster, as stated in the following quotes:

*P7: “to make sure that you have all emergencies stock in stock. You, your staff, must know what is going on.”*

*P8: “Disaster preparedness I think if something would happen that you make sure that whatever is necessary is in place like your fire extinguishers and stuff like that.”*

In Theme One, the participants conceptualize disaster as a sudden disruption in the routine. Many participants refer to physical disasters such as fire, flood, and gas explosions, few list pandemics. The participants acknowledged that a disaster plan must be implemented to manage the unknown. Although the participants demonstrated an understanding of the concept of disaster plans, most participants needed clarification on its location and content. Even though the nurses demonstrate some understanding of their roles and responsibilities during a disaster, the researcher observed a lack of assertiveness in other participants. Some participants needed clarification about the disaster plan (participants 5,10,14,15). According to Aykan et al. [23], for nurses to be adequately disaster-prepared, they require technical competencies such as scientific knowledge, disaster experience, and disaster exercises.

### Theme 2: nurses attitudes towards disaster preparedness in an obstetric unit

Participants appear to be well-informed about the concept of a disaster plan and have described the shortfalls of preparedness within the unit. Two sub-themes emerged; lack of preparedness when disaster strikes and awareness of disaster plans, as discussed below.

#### Sub-theme 2.1: lack of preparedness when disaster strike

The findings demonstrated that the obstetric unit is unprepared for a disaster. Very few rated to rate a full ten when enquired about the elements needed for preparation. The perceptions of the respondents are evident in the following quotes:

*P8: “I think I would think we’d go for a 5, because some of the management doesn’t actually bother”, “there should be stuff in place, but it’s not always in place”.*

*P13: “If I have to think of everyone being informed. I would say like at 8, OK.”*

Other participants [8, 10, 13, 15, 17–19] expressed feelings of unpreparedness by rating less than five:

*P10: “OK, since we only have exit plan I think I will give it a 1 (one). Because we’re now going to run with this exit plan and that’s it. That’s all I can give.”*

*P5: “I can’t speak for day shift, but like my night shift- No. Uhm, two. We’re not ready.”*

*P14: “Like previously. I will say. Three to four. Like I said, if we didn’t. If we didn’t participate in a drill yet, so it’s difficult if a disaster strike.”*

#### Sub-theme 2.2: awareness of disaster plans will contribute to disaster preparedness

Planning is the golden key for healthcare providers to respond effectively to a disaster [24], which includes disaster plans for healthcare workers to know how to respond, communicate, recognize one’s responsibilities, and acknowledge the plan’s contents.

Participants verbalize that knowing about the disaster plan will prepare staff to ensure staff and patient safety. The following quotes support this:

*P1: “I think I should know because it is important because it is for the patient safety, and it is for your safety as well.” “Because you must also know what could be done in such a situation.”*

*P2: “Yes, because we put other people’s life in our hands firstly. And we are responsible to them as long as you are here, and no place can function without the disaster plan.”*

In addition, participants strongly agree on a team- approach to disaster management. Participants mentioned that the involvement of other staff in the hospital and a multi-disciplinary approach will also result in more successful disaster planning.

*P10: “The CEO, all the managers and everybody inside the ward of every ward from this. Matron to the cleaner. Every category is supposed to know what their position is supposed to be. If there is a disaster.”* [Chief Executive Officer].

Participants have demonstrated awareness of managing potential risks and hazards in their working environment.

*P8: “Most probably be patient smoking because we do get patient smoking because there’s oxygen points. A fire. A flood. Or even a pipe would burst, and the patients is there.”*

Participants acknowledged that disaster training should be compulsory for all staff to stay updated with the latest disaster management developments. Participants [2–4, 8, 12] expressed that it is essential for all nursing staff to attend training and that all should be included, including the different shifts (night versus day).

*P2: “Training is for all of us and that’s why I included the kitchen staff, the porters, the securities. Because they’re all part of our staff in the ward.”*

Participants [1, 4, 8, 9, 12, 13] agreed that a six-monthly update of the disaster plan should be implemented to ensure all personnel are regularly updated.

P1: “I can say six months.”

*P1: “Because twice a year, they must remind so that we know what’s going on, because other things there is update, maybe we still have the old information now there is a new information.”*

Furthermore, participants recognized that the likelihood of a potential disaster cannot be ignored. Some participants have reflected on their experiences of encountering a disaster within their work environment. This is evident in the following quotations:

*P2: “Yes, there was one time that we had actually the sister was in theatre, and then the equipment start burning. So, it just tells you how quickly a disaster can happen.”*

P10: “The roof did collapse on us one night.”

*P12: “The other day I was fetching a baby from theatre and then I was resuscitating the baby on the open incubator in theatre and then fire exploded just out of the blue.”*

Participants also indicated that disaster simulations should occur frequently within the hospital and the obstetric ward.

P7: “*At least every six months, there should be a mock drill. We have done that before, maternity never had a chance” “but it must be done.”*

P8: *“I think it’s like maybe if you can do it once a month with each shift. Day and night would actually be smart.”*

In Theme Two, the nurses’ attitudes towards disaster preparedness indicate certain elements which lack thorough preparation. The participants demonstrate that awareness of disaster plans will ensure patient and staff safety. The participants acknowledge that disaster management is a team approach, and that training should be compulsory for all staff. The respondents have stated that a six-monthly update of disaster plans and frequent disaster drills will be a good practice to ensure effective disaster management.

### Theme 3: disaster practices in the obstetric unit

Frequent drills, rehearsals, and regular training are essential to ensure disaster readiness. An international study supports this [25]. Emergency preparedness exercises are an important part of emergency preparedness. Hospitals should conduct at least one communication exercise every six months, a desktop exercise once a year, and a major live exercise every three years [25] as part of their disaster plan. The respondents of the obstetric unit referred to their current disaster practices and experiences. Sub-themes include the current disaster practices of the obstetric unit, as illustrated below.

#### Sub-theme 3.1: current disaster practices

The study’s respondents [1–4, 8, 9, 13, 15, 19] explained that many have never participated in any disaster drill in their years of nursing. The following statements can support this:

*P4: “I’ve never seen a drill here, so maybe it was done, but I was never involved in one of the drills.”*

*P10: “we never had any drill. We don’t know of the plan. We don’t know of our such of your position when there’s a disaster”. “None. It doesn’t happen here. I’ve never seen it. In my 30 years. I don’t think the management thinks we’ll ever have a disaster.”*

*P16: “honest with you, I’m now here for eight years. I’ve never, ever took part in a disaster drill.”*

Respondents have mentioned a need for more training opportunities or certainty regarding training. This is evidenced below:

*P5: “I don’t think we have enough knowledge because we don’t go for training. We don’t.”*

P14: “I never went for training.”

*P15: “No. They’ve never done anything. It must be a forced thing. Everyone must be aware of it.”*

Participants have also expressed uncertainty regarding regular updates of disaster plans.

P5: “No, I don’t think they update it often.”

In Theme Three, disaster practices are explored. Participants confirmed the lack of disaster drills. Some respondents concede that formal training opportunities are not equally available and are still determining the regular updates of disaster plans. Literature supports the need for frequent training. Systematic disaster practices and readiness enhancement are essential to secure disaster preparedness, such as drills [26] and the implementation of sufficient training.

### Theme 4: essential key factors needed for successful disaster management in an obstetric unit

Participants have demonstrated insight regarding essential factors required for successful disaster preparedness. Sub-themes include the factors contributing to the success of disaster management in an obstetric unit and factors impeding the success of disaster management in an obstetric unit, as outlined below.

#### Sub-theme 4.1: factors contributing to the success of disaster management in an obstetric unit

Respondents emphasized the importance of creating daily disaster consciousness by the allocation of roles and responsibility acknowledgement of the nurses and task division.

P2: *“If I’m the sister in charge in the ward I must make sure every morning that all my people know what is the basic plan for exit to perform in a plan of evacuation. If we have babies in a nursery, the nurse must know exactly what to do.”*

Participants [4, 8, 10, 11] agreed that regular updates on the number of patients in the ward at a specific time several patients are critical to ensure successful disaster management.

*P8: “That’s why we do the six hours stats”.*

Furthermore, participants [3, 9, 12, 14, 16, 17, 19] acknowledged that a fully functional communication system is a requirement in successful disaster management.

*P13: “I just feel that there should just be communicated thoroughly with everyone often enough about what’s going on. Especially on all shifts.”*

The interviewees mentioned that successful disaster management requires strong leaders.

*P9: “We are prepared now because we do have the leaders who can lead us when the disaster come up.”*

Participants emphasized that sufficient disaster management equipment, such as fire extinguishers and emergency trolleys, must be in place.

*P16: “Fire extinguisher. I must know how to use. There are emergency trolleys. Those are in place.”*

In Theme 4.1, participants mentioned factors contributing to successful disaster management in the obstetric unit, such as the daily allocation of disaster management roles and daily disaster consciousness. The respondents also listed regular updates of patient statistics, effective communication, strong shift leaders as necessary factors, and sufficient resources (fire extinguishers and emergency trolleys) to be in place.

#### Sub-theme 4.2: factors impeding the success of disaster management in an obstetric unit

The participants demonstrated awareness of impeding factors towards disaster management in an obstetric unit. These impeding factors are outlined.

Respondents [8, 10, 11, 13, 17] perceived themselves ineffectively prepared for disasters due to uncertainty regarding their roles and a lack of exposure during disaster management. This is illustrated below:

*P5: “I don’t think we have enough knowledge because we don’t go for training. We don’t. Some of us don’t even know what is our roles.”*

*P14: “I have worked here for three years, and I haven’t gone for training or I was not even involved in a drill.”*

Participants (1 to 12) emphasized inadequate resources, such as equipment and supplies, as well as the staff shortage, as impeding factors to the success of disaster management.

*P1: “The staff side there is a shortage. Nurses are not enough. Run around for wheelchairs. It’s not enough.”*

Furthermore, participants [2–4, 12, 14] mentioned that high patient acuity is an impeding factor to successful disaster management.

*P9: “So I think my problem is only on that we have facing a huge amount of the patient while we are less staff.”*

*P11: “we’re overflowing with patients and we’re busy”.*

Interviewees reported special requirements for vulnerable patients may impede successful disaster management. This is denoted by the following comments:

*P3: “So now if that were to happen in our nursery, we need to consider will we be able to bag our babies on the ventilators with CPAP or whatever. Because on that side we have more CPAPs. They are dependent on oxygen.”* [Continuous Positive Airway Pressure].

*P7: “That is my biggest thing is how to get a mother in labour out and then babies on CPAP.”*

Respondents have indicated that uninterrupted obstetric care could negatively affect the effective evacuation during a disaster:

P2: “It cannot be postponed. It has to happen.”

*P3: “So if you do resus on a baby, well, you can. You can try to bag (mechanically ventilate) the baby out. Still resus the baby out, but in case of an of a delivery that is a problem because you can’t go on with the delivery.”*

Participants have stated that renovations at the facility cause insecurity on how to execute the disaster management plan.

*P9: “The disaster plan of this hospital, since that we are in the chaos of the renovation now we are a little bit confused about the things.”*

Additionally, participants also mentioned the concern regarding exits, limited access control of one remote and cluttered passages.

*P4: “We only have one door at the back of which sometimes you don’t have access. You don’t have a remote for this door, and it’s too far to go that side. Even the passage is full of cots. You need to remove the stuff because you can’t move.”*

In Theme 4.2, the participants acknowledged the impeding factors of disaster management in the unit, such as uncertainty regarding responsibilities. Participants perceived resources, equipment, supplies, and staff attendance as inadequate. The high patient turnover is a concern to respondents. The respondents also raised concerns about requirements and the uninterrupted essential obstetric care that could hamper effective disaster management practices. The facility’s renovation is another concern because the cluttered passages may obstruct evacuation.

### Theme 5: recommendations to ensure successful disaster management in the obstetric unit

In Theme Five, respondents identified shortfalls of the obstetric unit relating to successful disaster preparedness and verbalized recommendations to improve disaster management. Recommendations included staff involvement and acknowledgement of their inputs in disaster plan development, user-friendly disaster plans and regular in-service training and updates. As discussed below, a sub-theme includes strategies to improve disaster awareness and preparedness in the obstetric unit.

#### Sub-theme 5.1: strategies to improve disaster awareness and preparedness in the obstetric unit

Participants demonstrate an understanding of ways to improve disaster preparedness within the obstetric ward.

P5: “More training about being prepared. Drills for us on the night.”

*P11: “Communicating is the first thing. And to be. Regularly prepared, be prepared for a disaster. And we need to practice at it.”*

Participants reported the involvement of staff and their input in developing a disaster plan to ensure successful disaster management.

*P11: “Everybody that’s working in the hospital. They have to have an input in this. Because for it to be successful you need everybody.”*

The participants emphasized that regular in-service training and frequent updates should be performed.

*P9: “They need to organise those people to come and do the in-service training around the facilities.” “They must make sure so there must not be any staff member who left behind in terms of their knowledge.”*

Participants have also recommended suggestions for user-friendly disaster plans.

*P8: “You can do your pictures system and even your arrows to show with your pictures to show which way to go to if this happens. And obviously educate the patients.”*

Theme Five explored recommendations to ensure successful disaster management in the unit. These included ways to improve awareness among staff and patients, involvement of staff and their inputs in developing a disaster plan, regular in-service training and frequent updates, and user-friendly disaster plans.

## Discussion

The study’s findings correlate with literature emphasizing disaster preparedness’s importance. The study of Al Harthi et al. [27] explains that it is critical to identify the challenges of disaster management in nursing for the improvement and development thereof and for evidence-based practice progression. Schumacher et al. [28] indicate that gaps in preparedness could magnify the adverse effects of an unexpected disaster, and thus, hospitals should actively improve their guidelines and disaster preparedness.

Similar to the results asserted by Al Harthi et al. [27], the study participants mentioned unfamiliar with the unit disaster plan and the disaster policies. Others suggested that it could be advantageous to be involved in the development of the design of a disaster plan, as this could enhance their familiarity with the disaster plan and policy. Similarly, Barfied and Krug [29] referred to the importance of NICU personnel participating in their facility’s disaster-planning activities and disaster simulations.

Globally, nurses still have low to moderate levels of disaster preparedness in knowledge and skill competencies. Thus, more improvements need to occur to assure disaster preparedness [30]. Some participants reported being assertive during a disaster. However, others felt they may need to learn what to do and are unfamiliar with the disaster plan. Lack of disaster plans and perceptions of unpreparedness may lead to chaos and complete disorientation, as Goniewicz and Goniewicz [31] reported. Tas and Cakir [32] asserted that training and education programs should be regularly employed to strengthen the knowledge, awareness, and skills of disaster preparedness nurses. Participants expressed the need to create daily disaster consciousness and awareness in staff and patients. Participants recommended that more frequent disaster simulations be offered, which was mentioned by Skryabina et al. [33], who indicated that disaster exercises are practical for improving participants’ knowledge of emergency tasks, policies, and procedures. Moreover, drills and exercises improve confidence, competence, and understanding of individual and team roles.

Al Harthi et al. [27] also mention that an effective disaster plan needs to address surge capacity, staff, and resource shortages. As many of the participants raised concerns about the staff and resource shortage, the disaster plan should include solutions or alternatives to these situations [27]. Monteblanco and Leyser-Whalen [34] explained that the influx of patients into a system that cannot accommodate them is the very definition of a disaster because the event exceeds the response capacity of available resources. The participants mentioned the overflow and the high nurse-to-patient ratio as concerns. The participants expressed their concern about how to deal with vulnerable patients during a disaster, such as patients actively in labor or critically ill infants on CPAP. According to Abir and Daniels [35], pregnant and peripartum populations require tailor-made disaster plans that specify care provision for a wide range of acuity levels.

The responses of the participants also stressed the critical evaluation of resources and the need for improved clarity and organization during a disaster. These responses are supported by international literature. A study by Sarma et al. [36] emphasizes the importance of effective coordination of disaster resources, effective planning for a multicriteria decision-making process and preparedness for improved organization and distribution during a disaster.

## Recommendations

Recommendations to the obstetric unit include ways to improve disaster awareness among staff and patients:

- Allocate a person on every shift to be the primary person to take the lead in case a disaster should occur;
- Increase the collaborative contribution of all personnel within the department to formulating the emergency preparedness strategy and regulations.
- Increase the frequency of educational and training opportunities and make these compulsory for all to attend;
- Regular in-service training should be instilled, such as spot training;
- All staff members should be included in a disaster drill or evacuation rehearsal at least twice a year.
- Ensure that the disaster plans are always available and visible.

## Conclusion

The need for hospitals to establish and maintain adequate disaster preparedness measures is paramount considering the rising frequency of worldwide disasters, as they directly impact human survival. Specialized care during disaster events should be extended to obstetric and neonatal populations, recognizing their vulnerability. Consequently, developing specific recommendations tailored to obstetric units’ unique needs is imperative. In order to enhance obstetric care and mitigate obstetric morbidity and mortality on a global scale, it is imperative to achieve effective disaster preparedness in obstetrics units. Furthermore, further study is necessary in this domain to address existing gaps in knowledge.

This explorative, descriptive qualitative study aimed to contribute to the knowledge on obstetric disaster preparedness and explore obstetric nurses’ knowledge and attitudes, on disaster preparedness. The study findings indicate that more education and training opportunities should regularly be available to the study population. More disaster drills and simulation exercises should be implemented to ensure confidence in disaster preparedness. Overall, the involvement of the obstetric staff in policymaking and disaster plan development is essential. Future research implications may link to a more extensive study of population and government hospitals of different levels of care, such as tertiary institutions or secondary levels hospitals.

### Electronic supplementary material

Below is the link to the electronic supplementary material.

Supplementary Material 1

## Data Availability

No datasets were generated or analysed during the current study.

## Declarations

### Limitations of the research

This was a qualitative study to explore the disaster preparedness of nurses in a specific obstetric unit of only one hospital. Even though the researcher obtained data saturation, the sample population was relatively small. Little to no information on disaster preparedness within obstetric units in South African government hospitals are available and thus the researcher had no guidance of prior research or literature as referencing framework.

## Abbreviations

CEO: Chief Executive Officer
CPAP: Continuous Positive Airway Pressure
EDQ: Contextual, exploratory, descriptive qualitative
KMC: Kangaroo Mother Care
MPNH: Maternal, Perinatal and Neonatal Health Policy

## References

[1] World Health Organization & EcoHealth Alliance (WHO & EHA). Disasters and emergencies: definitions training package. 2002. https://apps.who.int/disasters/repo/7656.pdf Accessed 14 May 2022.

[2] Ortiz-Barrios M, Gul M, López-Meza P, Yucesan M, Navarro-Jiménez E. Evaluation of hospital disaster preparedness by a multi-criteria decision making approach: the case of Turkish hospitals. Int J Disaster risk Reduct. 2020;49. 10.1016/j.ijdrr.2020.101748 10.1016/j.ijdrr.2020.101748 PMC7335495 32834973

[3] Farah B, Pavlova M, Groot W. Hospital disaster preparedness in sub-saharan Africa: a systematic review of English literature. BMC Emerg Med. 2023;23(71). 10.1186/s12873-023-00843-5 10.1186/s12873-023-00843-5 PMC10291806 37365529

[4] Ayenew T, Tassew SF, Workneh BS (2022). Level of emergency and disaster preparedness of public hospitals in Northwest Ethiopia: a cross-sectional study. Afr J Emerg Med.

[5] Shrivastava P (2002). Principles of Emergency Planning and Management. Risk Manage.

[6] Australian Institute for Disaster Resilience. Australian Disaster Resilience Knowledge Hub -Glossary. 2017. https://knowledge.aidr.org.au/glossary/?wordOfTheDayId=&keywords=α=R&page=2&results=50ℴ=AZ. Accessed 24 April 2024.

[7] World Health Organization. Hospital safety index: a guide for evaluators. 2015. Retrieved 26/09/2023 from https://iris.who.int/bitstream/handle/10665/258966/9789241548984-eng.pdf?sequence=1&isAllowed=y. Accessed 24 April 2024.

[8] Sahoo KC, Negi S, Patel K, Mishra BK, Palo SK, Pati S (2021). Challenges in maternal and child health services delivery and access during pandemics or public health disasters in low-and middle-income countries. Healthcare.

[9] Samsuddin NM, Takim R, Nawawi AH, Alwee SNAS (2018). Disaster preparedness attributes and hospital’s resilience in Malaysia. Procedia Eng.

[10] Hodge AJ, Miller EL, Skaggs MKD (2017). Nursing self-perceptions of emergency preparedness at a rural hospital. J Emerg Nurs.

[11] Kingsley JP, Vijay PK, Kumaresan J, Kumaresan J, Sathiakumar N (2021). The changing aspects of Motherhood in Face of the COVID-19 pandemic in low- and Middle-Income Countries. Matern Child Health J.

[12] Boyles AL, Beverly BE, Fenton SE, Jackson CL, Jukic AZ, Sutherland VL, Baird DD, Chandler KJ (2021). Environmental factors involved in maternal morbidity and mortality. J Women’s Health.

[13] Statistics South Africa (Stats SA). South Africa Demographic and Health Survey 2016 Key Findings. 2017. https://dhsprogram.com/pubs/pdf/SR248/SR248.pdf. Accessed 16 August 2023.

[14] Ngobeni V, Breitenbach MC, Aye GC. Technical efficiency of provincial public healthcare in South Africa. BMC J Cost Eff Resource Allocation. 2020;18(3). 10.1186/s12962-020-0199-y 10.1186/s12962-020-0199-y PMC6986147 32002018

[15] Suzuki E, Kouame C, Mills S. Progress in reducing maternal mortality has stagnated and we are not on track to achieve the SDG target: New UN report. 2023. https://blogs.worldbank.org/opendata/progress-reducing-maternal-mortality-has-stagnated-and-we-are-not-track-achieve-sdg-target. Accessed 9 September 2023.

[16] Department of Health [DoH]. South African Maternal, Perinatal, and Neonatal Health Policy. 2021. https://knowledgehub.health.gov.za/elibrary/south-african-maternal-perinatal-and-neonatal-health-policy. Accessed 26 February 2023.

[17] Sharma SK, Rani R (2020). Nurse-to-patient ratio and nurse staffing norms for hospitals in India: a critical analysis of national benchmarks. J Family Med Prim care.

[18] Saunders M, Lewis P, Thornhill A (2023). Research methods for business students.

[19] Stander M. Hospital disaster planning in the Western Cape: Are we ready for 2010? [Dissertation- MA]. Cape Town: UCT. 2008.

[20] Moabi MR. Knowledge, attitudes and practices of health care workers regarding disaster preparedness at Johannesburg hospital in Gauteng Province, South Africa. [Dissertation- MPH]. Johannesburg: University of the Witwatersrand. 2008.

[21] Caulfield J. How to Do Thematic Analysis | A Step-by-Step Guide & Examples. 2022. https://www.scribbr.com/methodology/thematic-analysis/. Accessed 16 May 2022.

[22] Braun V, Clarke V (2006). Using thematic analysis in psychology. Qualitative Res Psychol.

[23] Aykan EB, Fidancı BE, Yıldız D. Assessment of nurses’ preparedness for disasters. Int J Disaster Risk Reduct. 2022;68. 10.1016/j.ijdrr.2021.102721

[24] Al-Thobaity A, Alamri S, Plummer V, Williams B. Exploring the necessary disaster plan components in Saudi Arabian hospitals. Int J Disaster Risk Reduct. 2019;41. 10.1016/j.ijdrr

[25] Skryabina EA, Betts N, Reedy G, Riley P, Amlôt R. The role of emergency preparedness exercises in the response to a mass casualty terrorist incident: a mixed methods study. Int J Disaster Risk Reduct. 2020;46. 10.1016/j.ijdrr.2020.101503 10.1016/j.ijdrr.2020.101503 PMC7709486 33312855

[26] Mahdi SS, Jafri HA, Allana R, Battineni G, Khawaja M, Sakina S, Agha D, Rehman K, Amenta F (2023). Systematic review on the current state of disaster preparation Simulation exercises (SimEx). BMC Emerg Med.

[27] Al Harthi M, Al Thobaity A, Al Ahmari W, Almalki M (2020). Challenges for nurses in Disaster Management: a scoping review. Risk Manage Healthc Policy.

[28] Schumacher L, Senhaji S, Gartner BA (2022). Full-scale simulations to improve disaster preparedness in hospital pharmacies. BMC Health Serv re.

[29] Barfield WD, Krug SE. Disaster preparedness in neonatal intensive care units. Am Acad Pediatr. 2017;139(5). 10.1542/peds.2017-0507 10.1542/peds.2017-0507 28557770

[30] Said NB, Molassiotis A, Chiang VCL. Psychological preparedness for disasters among nurses with disaster field experience: an international online survey. Int J Disaster Risk Reduct. 2023;46. 10.1016/j.ijdrr.2020.101533

[31] Goniewicz K, Goniewicz M (2020). Disaster preparedness and professional competence among Healthcare providers: Pilot Study results. Sustainability.

[32] Tas F, Cakir M. Nurses’ knowledge levels and preparedness for disasters: a systematic review. Int J Disaster Risk Reduct. 2022;80. 10.1016/j.ijdrr.2022.103230

[33] Skryabina E, Reedy G, Amlôt R, Jaye P, Riley P (2017). What is the value of health emergency preparedness exercises? A scoping review study. Int J Disaster Risk Reduct.

[34] Monteblanco A, Leyser-Whalen O (2019). Thinking outside of the hospital and nurse-midwife paradigms: a qualitative examination of midwifery in Times of Natural disasters. Int J Mass Emergencies Disaster.

[35] Abir G, Daniels K. Multidisciplinary disaster planning for obstetrics. 2019. https://www.apsf.org/article/multidisciplinary-disaster-planning-for-obstetrics/. Accessed 16 August 2023.

[36] Sarma D, Das A, Dutta P, Bera UK (2022). A cost minimization resource allocation model for Disaster Relief Operations with an information crowdsourcing-based MCDM Approach. IEEE Trans Eng Manage.

[37] World Medical Association. WMA Declaration of Helsinki – Ethical principles for medical research involving human subjects. 2023. https://www.wma.net/policies-post/wma-declaration-of-helsinki-ethical-principles-for-medical-research-involving-human-subjects/. Accessed 28 December 2023.

