# Supplementary material for: Exploring disaster preparedness in an obstetric unit in a district hospital in the Western Cape Province
Source: BMC Health Serv Res. 2024 May 21;24:654. doi: 10.1186/s12913-024-11104-x (PMC11110375; doi:10.1186/s12913-024-11104-x)
Supplement: Supplementary file 1 — Supplementary Material 1 [file 12913_2024_11104_MOESM1_ESM.docx]

Additional file 1

Interview guide: Guidance for the researcher to be used in individual interviews.

Semi-structured Interview guide

Section 1: Demographics

1.State category of staff and level of experience:

1 – 5 years

5 – 10 years

10 – 20 years

20 – 30 years

2. How long have you worked in this hospital?

Section 2: Knowledge

3. What is a disaster?

4. What is a disaster plan?

5. Please direct me to find the disaster plan.

6. Please define disaster drills are?

7. Please list your functions during a drill.

8. What is disaster preparedness?

Section 3: Attitude

9. Do you think you need to know about disaster plans and why.

10. Rate the scale of which you think management is adequately prepared should a disaster occur and motivate.

11. How many people do you think are to be involved in disaster planning in the hospital?

12. Could you identify potential hazards likely to cause disaster should be identified and what would you recommend dealing with them.

13. Who do you think training is necessary for?

14. Do you think it is necessary to have a disaster plan and why?

15. How often do you think disaster plans need to be updated?

16. Do you think disasters are unlikely to happen in this hospital and why?

17. Do you think disaster management is for nurses and doctors only or which other staff?

18. Do you think disaster simulations should occur frequently in the hospital and how often or why?

Section 4: Practice

19. Are disaster drills done at your hospital and which type of drills are done?

20. How often does the hospital provide ongoing training for disaster preparedness?

21. When does the hospital update the disaster plan periodically and do you think this is often enough?

Questions self-created by the researcher (Not Moabi as source)

22. How effectively prepared is the obstetric unit of selected hospital in the Western Cape prepared for a disaster?

23. Are the nurses in an obstetric unit in a selected government hospital in the Western Cape prepared for their roles of disaster management?

24. Are the essential key factors for successful disaster management in place within obstetric units in selected government hospitals within the Western Cape?

25. What are the ways in which disaster preparedness can be improved within the obstetric unit in selected hospital in the Western Cape to be more effective?

26. What are the considerations to be rendered with regards to the obstetric patients and the neonatal infants?

27. What would you suggest for user friendly disaster plans, and evacuation plans that need to be visible at times and should also consider mothers who cannot read and write?

28. Are wheelchairs able to be sufficient and accessible to evacuate or mobilize those patients that cannot walk such as those mothers who have undergone Caesarean sections in the postnatal department?

29. Do you consider the nursing staff to be updated and accounted for at all times of the exact number of patients within the unit so that none is left behind in the event of an evacuation?

30. Would you agree that in the occurrence of disaster, obstetric care (such as active delivery or infant care) cannot be postponed and still needs to be implemented and practiced effectively?

31. Do you think the obstetric unit of a selected government hospital in the Western Cape is successfully prepared to execute their disaster plans when disaster strikes?
